# Diagnostic Accuracy of Cell Block Preparations and Clinical Features Affecting It in Vitreoretinal Lymphoma

**DOI:** 10.3390/jcm11051391

**Published:** 2022-03-03

**Authors:** Satoru Kase, Kenichi Namba, Daiju Iwata, Kazuomi Mizuuchi, Kayo Suzuki, Takako Ito, Keitaro Hase, Nobuyoshi Kitaichi, Susumu Ishida

**Affiliations:** 1 Department of Ophthalmology, Faculty of Medicine and Graduate School of Medicine, Hokkaido University, Sapporo 060-8638, Japan; kaseron@med.hokudai.ac.jp (S.K.); d.iwata@med.hokudai.ac.jp (D.I.); mizuuchi@peach.plala.or.jp (K.M.); ksuzuki@med.hokudai.ac.jp (K.S.); takako.gerbera@gmail.com (T.I.); k.hase59@med.hokudai.ac.jp (K.H.); nobukita@hoku-iryo-u.ac.jp (N.K.); ishidasu@med.hokudai.ac.jp (S.I.); 2 Department of Ophthalmology, Health Sciences University of Hokkaido, Sapporo 061-0293, Japan

**Keywords:** vitreoretinal lymphoma, cell block preparations, subretinal infiltrates, optical coherence tomography

## Abstract

Purpose: The purpose of this study was to examine the diagnostic accuracy of the cell block (CB) method and clinical features affecting it in patients with vitreoretinal lymphoma (VRL). Methods: This study enrolled 38 eyes in 33 VRL patients, and 7 eyes in 7 patients with idiopathic uveitis who underwent diagnostic vitrectomy. Medical records including the results of CB cytology, interleukin (IL)-10/-6 concentrations, and immunoglobulin heavy chain gene (IgH) rearrangement were retrospectively searched. Results: Patients with VRL comprised 16 women and 17 men, and the age of onset ranged from 44 to 85 years (mean: 70 years). CB preparations detected large malignant cells in 35 eyes (92%), whereas the other 3 VRL eyes were negative. Two of the latter three eyes showed subretinal infiltrates, which existed in 7 of 35 CB-positive eyes. Intravitreal IL-10 and -6 concentrations were 1866 ± 4088 pg/mL and 98 ± 139 pg/mL, respectively, and the rate of IL-10/-6 >1 was 86.9%. The presence of IgH monoclonality was 63.2%. In patients with uveitis, CB specimens revealed no atypical but small inflammatory cells. IL-6 concentration was 311.1 ± 240 pg/mL, whereas IL-10 was undetectable in six eyes, and the IL-negative rate was 85.7%. Six eyes (85.7%) with uveitis showed no IgH monoclonality. Conclusions: Diagnostic accuracy of CB preparations in VRL could achieve an equivalent outcome to IL ratio calculation and IgH monoclonality detection. The appearance of subretinal infiltrates may diminish the CB positivity.

## 1. Introduction

Intraocular lymphoma is a vision- and life-threatening intraocular tumor. The lymphomas can be divided into primary vitreoretinal lymphoma (PVRL), vitreoretinal lymphoma (VRL) from primary central nervous system lymphoma (PCNSL), and intraocular metastasis by systemic malignant lymphoma (secondary lymphoma). Clinically, patients with VRL commonly demonstrate vitreous haze as well as subretinal infiltrates [1]. When the patients have medical histories such as PCNSL or systemic lymphoma, the diagnosis of VRL may not be difficult in selected patients. For ophthalmologists, the diagnosis of PVRL is the most challenging overall because there are no specific clinical findings corresponding to PVRL. Therefore, pathological tests using the vitreous are required to make a correct diagnosis. Identification of malignant cells by cytological examination has been the most convincing evidence for diagnosis of VRL [2]; however, the diagnostic rates by cytological examinations are not high based on multicenter analyses in Japan [3]. That is why other pathological tests including intravitreal interleukin (IL)-10/-6 concentrations and immunoglobulin heavy chain gene (IgH) rearrangement combined with cytology might be recently considered a new stringent gold standard for diagnosis of VRL [4] and are indeed helpful given the low cytological positivity [5].

In order to overcome the low positive rates in cytological examinations, several reports have shown that cell block (CB) preparations from the vitreous taken at vitrectomy contributed to favorable cytological diagnostic rates in VRL patients [6,7,8]. CB preparations using shed vitreous aspirates under infusion have several advantages such as collecting many cells compared to undiluted anterior vitreous fluids, immunohistochemical examinations available beyond morphological evaluation, and further genetic tests [9]. In fact, we showed that the cytological diagnostic rate was higher in the CB preparation method than in conventional smear cytology [10]. However, little is known about differences in sensitivity and specificity between the CB method and other pathological tests. On the other hand, Ito et al. recently reported risk factors associated with the failure of cytological diagnosis using CB preparations [11]. Even though the cytological positivity using the CB method would improve, it remains unknown how CB-negative VRL eyes are diagnosed and eventually managed. With recent advances in imaging modalities, optical coherence tomography (OCT) is likely to play a critical role in the early diagnosis of VRL. Subretinal infiltrates, actually often detected as subretinal pigment epithelial (RPE) deposits on OCT, and hyper-reflective lesions within the retina are common findings in VRL [12]; however, there are few reports on how OCT findings affect the diagnostic accuracy in VRL patients who tested negative for pathological assessment using CB specimens.

The aim of the present study is to examine the sensitivity and specificity of the CB preparation method to evaluate the accuracy of diagnosing VRL in comparison with IL and IgH tests and to search for clinical features affecting the CB-based diagnostic accuracy in VRL patients.

## 2. Materials and Methods

This is a retrospective observation study. The institutional review board (IRB) approved this study design and use of human materials (IRB number: 019-0108). All the patients underwent diagnostic vitrectomy at Hokkaido University Hospital. This study enrolled 38 eyes of 33 patients definitely diagnosed with VRL, and 7 eyes in 7 patients diagnosed with idiopathic uveitis, from January 2012 to December 2018. Medical records including ophthalmological findings such as best-corrected visual acuity, intraocular pressure (IOP), slit-lamp examination, funduscopic examination, the B scan of OCT, laboratory tests, and pathological findings in the vitreous, as well as results of imaging modalities such as brain magnetic resonance imaging (MRI) and positron emission tomography–computed tomography, were searched.

Cytological examinations as well as immunocytochemistry with anti-CD3 and CD20 antibodies were conducted with CB preparations as described previously [9,10]. Briefly, undiluted vitreous fluids were obtained during anterior vitrectomy without infusion for the measurement of IL-10/-6 concentrations. Then, shed vitreous aspirates under infusion were harvested following core vitrectomy with a 25-gauge needle and were centrifuged, and cellular pellets were submitted for CB and IgH tests. Diagnosis of VRL was made based on ophthalmological findings, including OCT findings such as subretinal/sub-RPE infiltration, as well as responsiveness to intravitreal methotrexate injections (IV-MTX); OCT findings such as sub-RPE infiltration [12] before vitrectomy or treatments for VRL; and the results of pathological tests including cytology, IL-10/-6 concentrations determined by conventional enzyme-linked immunosorbent assay (ELISA), and monoclonal IgH rearrangement detection. When malignant lymphoma cells were definitely confirmed in CB preparations, the cytological findings were evaluated as positive for the diagnosis of VRL. Even when the CB results were not consistent with lymphoma, patients positive for other multiple findings of IL-10/-6, IgH, OCT, and responsiveness to treatments were also diagnosed with VRL in this study. When the IL-10/-6 ratio was more than 1 and/or IL-10 concentration was over 100 pg/mL, the results of IL were evaluated as positive for a VRL diagnosis. If monoclonal IgH rearrangement of variable framework region at joining (JH) region was confirmed by polymerase chain reaction, the IgH results were considered positive for a VRL diagnosis. 

The patients with idiopathic uveitis underwent diagnostic vitrectomy, since ocular findings were suspicious for VRL due to vitreal haze, in addition to no specific findings of serum and urinary tests as well as chest *X*-ray, which did not reach definite diagnoses of uveitis such as sarcoidosis, Vogt–Koyanagi–Harada disease, Behçet’s disease, infectious endophthalmitis, and acute retinal necrosis. The treatment regimen for VRL patients in this study was basically a weekly dosage of IV-MTX for 8 weeks at a dose of 400 μg in 0.1 mL from the pars plana using a 30-gauge needle. The patients occasionally further underwent IV-MTX every month until the vitreal haze and/or subretinal infiltrates diminished. Selected patients received systemic chemotherapy, including an intravenous high-dose MTX injection, and/or whole-brain radiation therapy.

### Statistical Analysis

The Fisher’s extract test was employed to evaluate the difference in CB positivity between VRL eyes with and without subretinal infiltrates. A *p*-value of less than 0.05 was considered to indicate a statistically significant difference.

## 3. Results

In VRL, 33 patients consisted of 17 males and 16 females. The mean age at the onset of ocular symptoms was 70 years (44–86 years). Among 33 patients, 16 patients were classified as PVRL since lymphoma lesions were not detected in the other organs upon diagnosis with VRL. In contrast, the other 17 patients had intraocular involvements due to PCNSL/systemic lymphoma. 

**Figure 1:**
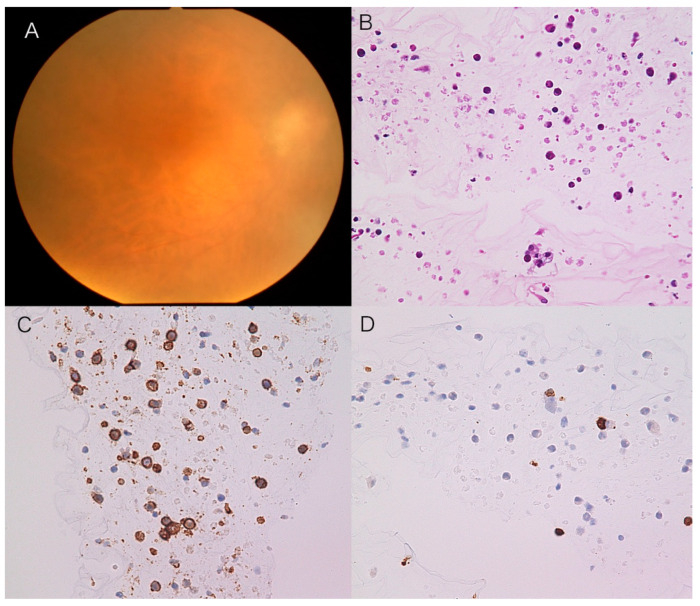
Representative case with vitreoretinal lymphoma showing positive cell block cytology. Fundus shows 2+ vitreal haze OD (**A**). The CB specimen shows large malignant cells in the vitreous (**B**) with CD20-positive cells (**C**) where CD3-positive small T cells are intermingled (**D**).

Malignant cells were cytologically detected in 35 eyes (92.1%) using CB preparations. Immunocytochemistry with CD3, a T-cell marker, and CD20, a B-cell marker, was conducted in all the patients examined. In CB-positive VRL cases, all the malignant cells were CD20-positive and CD3-negative (Figure 1). In three VRL cases that failed to show CB positivity, one patient had a medical history of PCNSL. Of the three CB-negative eyes, two and three cases were diagnosed with VRL based on IL concentrations and subretinal infiltrates on OCT, respectively. IgH monoclonality was noted in two of the three CB-negative cases.

OCT detected the abnormal reflectivity between the RPE layer and Bruch’s membrane corresponding to subretinal infiltrates. There were ophthalmoscopically obvious subretinal infiltrates in 9 of 38 eyes with VRL. Subretinal infiltrates were observed in only 7 of 35 CB-positive eyes, whereas two of three CB-negative eyes presented subretinal infiltrates. There was no statistically significant difference in CB positivity between eyes with and without subretinal infiltrates. All the OCT findings were resolved after treatments for VRL.

Intravitreal IL-10 and IL-6 concentrations were 1866 ± 4088 pg/mL and 98 ± 139 pg/mL, respectively. The IL-positive and IgH-positive rates were 86.8% (33 eyes) and 63.2% (24 eyes), respectively, in all the 38 VRL eyes. In contrast to the impact of subretinal infiltrates on CB positivity, subretinal infiltrates were almost evenly detected according to the results of IL and IgH tests, specifically in 9 of 33 IL-positive eyes versus 1 of 5 IL-negative eyes, as well as in 5 of 24 IgH-positive eyes versus 4 of 14 IgH-negative eyes. 

In seven patients (three men and four women) with idiopathic uveitis, the mean age was 61 years (47–90 years) when diagnostic vitrectomy was conducted. CB specimens detected small reactive lymphoid cells, whereas no atypical cells were observed in any specimens (0%). Intravitreal IL-10 concentrations were undetectable (<10 pg/mL) in six eyes, while IL-10 concentration was 130 pg/mL in one eye, being more than 100 pg/mL, evaluated as IL-positive. Intravitreal IL-6 concentrations were 311 ± 240 pg/mL in seven eyes, in which no eyes revealed an IL-10/-6 ratio greater than 1. Therefore, the IL-positive rate was 14.3% (one eye) in all the seven uveitis eyes. One eye, but not the IL-positive eye, tested positive for IgH monoclonality; however, the result is considered false-positive, which could reflect restricted B-cell populations found in uveitis, known as pseudo-monoclonality [4]. The uveitis case with IL-positive or IgH-positive results did not develop VRL so far at 21 months and 30 months, respectively, after the diagnosis without chemoradiotherapy. The sensitivity and specificity of CB, IL, and IgH tests are shown in Table 1. A representative CB-negative case with VRL is as follows:

### A Representative Case

**Figure 2:**
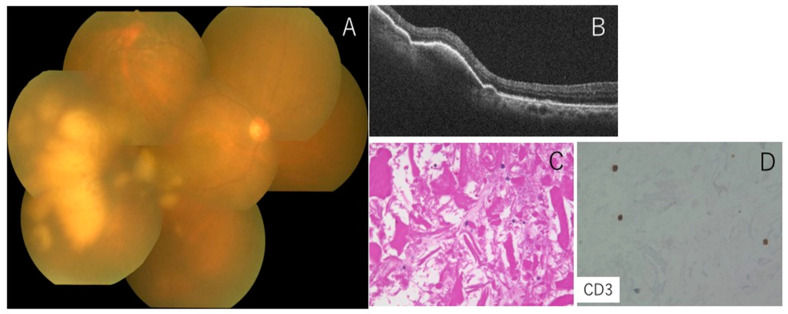
Representative case 1 with vitreoretinal lymphoma showing negative cell block cytology. Fundus shows 1+ vitreal haze and irregular subretinal infiltrates OD (**A**). OCT displays abnormal reflection between the RPE layer and Bruch’s membrane (**B**). The CB specimen shows no malignant cells in the vitreous (**C**), where CD3-positive small T cells are intermingled (**D**).

**Figure 3:**
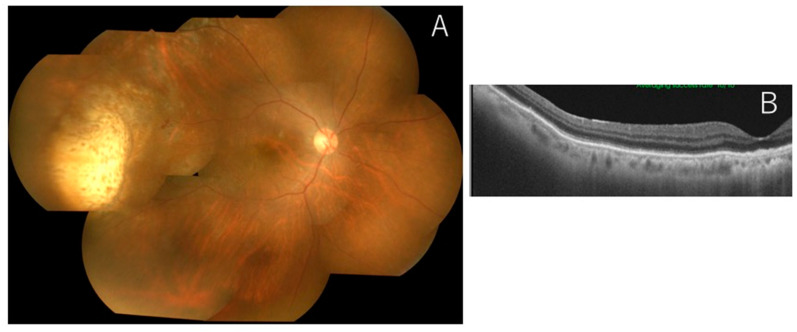
Representative case 1 after intravitreal methotrexate (IV-MTX) injection. After IV-MTX, the vitreal haze and subretinal infiltrates were resolved (**A**). Abnormal refection between the RPE layer and Bruch’s membrane disappeared following IV-MTX (**B**).

An 86-year-old female complained of blurred vision in her right eye, oculus dexter (OD). Past medical and family histories were nothing of note. She suffered from blurred vision OD two months prior and was referred to our hospital. Best-corrected visual acuity was 0.5 OU with normal IOP. Slit-lamp examination revealed mild inflammation in the anterior chamber (1+ flare, 2+ cells) with keratic precipitates. Fundus showed 1+ vitreal haze and irregular serpiginous subretinal infiltrates OD (Figure 2A). OCT displayed abnormal reflection between the RPE layer and Bruch’s membrane (Figure 2B). MRI demonstrated no abnormalities in the brain. CB specimens obtained during diagnostic vitrectomy did not detect malignant cells in the vitreous (Figure 2C), where CD3-positive small T cells were intermingled (Figure 2D). Further pathological analyses proved negative IgH monoclonality but showed a high concentration of IL-10 (650 pg/mL) compared to IL-6 (84 pg/mL). Although the CB method did not provide convincing evidence of VRL, the patient was diagnosed with VRL based on IL values and OCT findings. The patient received IV-MTX, resulting in the resolution of vitreal haze (Figure 3A) as well as subretinal infiltrates (Figure 3B). 

## 4. Discussion

In the present study, cytological diagnosis was conducted based on CB preparations, in which cell surface markers including CD20 and CD3 could be further examined. As a result, malignant cells were cytologically detected in the vitreous in 92.1% of the VRL eyes but none of the uveitis eyes using CB preparations, in which the sensitivity and specificity were 0.92 and 1.00, respectively. Positive rates in cytological diagnosis of VRL were not favorable when conventional smear cytology was applied to the undiluted vitreous samples [10,11]. The authors demonstrated that the diagnostic rates of VRL were about 2.5 times more favorable by CB preparations than those by conventional smear cytology in our institute [10]. In this study, cytological examination was conducted with CB preparations in all the patients, which possibly contributed to the better diagnostic rate of VRL. 

This study also revealed the IL sensitivity and specificity to be 0.87 and 0.86 as well as the IgH sensitivity and specificity to be 0.63 and 0.86, respectively. Cassoux et al. demonstrated the diagnostic significance of intravitreal IL-10 concentration greater than 400 pg/mL with the sensitivity and specificity being 0.99 and 0.80 [13]. Santos et al. demonstrated high sensitivity and specificity of cytology, cytokine, and flow cytometry analyses in a large number of patients with VRL [4]; however, data on flow cytometry were not available in this study. On the other hand, using the same criteria of IL positivity with our present study (IL-10/-6 ratio >1 and/or IL-10 concentration >100 pg/mL), Sugita et al. showed the sensitivity and specificity of 0.82 and 1.00, respectively, together with the IgH sensitivity and specificity of 0.95 and 1.00, respectively [14]. Our CB preparation method showed feasible sensitivity and specificity equivalent to those of IL and IgH results, indicating that the diagnostic accuracy of CB cytology compares favorably with other currently used pathological tests in VRL patients.

Despite the high diagnostic rate of CB cytology, three eyes with VRL tested negative for the CB preparation method in this study. All of these three eyes had vitreous haze. CB specimens revealed small lymphocytes without cellular atypia, suggesting that these patients’ vitreous hazes were caused by inflammation rather than intravitreal invasion of lymphoma cells, which were likely to be confined to subretinal/sub-RPE infiltrates detected funduscopically and on OCT. Indeed, as many as two of three CB-negative eyes presented subretinal infiltrates, whereas only 7 of 35 CB-positive eyes did so, showing some difference in CB positivity between eyes with and without subretinal infiltrates. Ito et al. reported risk factors for failure of CB-based diagnosis [10]. They reported that 26 of 35 eyes (74.3%) examined were negative for CB technique in VRL, and they concluded via multivariate analysis that subretinal infiltrates were most likely to have a negative impact on the diagnostic rate with CB specimens [10], in consistence with our present data showing that subretinal infiltrates could be a risk factor for failure of malignant cell detection in the vitreous.

Cytological detection of malignancy in the vitreous is the most reliable diagnostic finding in VRL patients [2]. With regard to starting treatments, it is disputable in clinical practice whether to manage VRL-suspicious patients who are CB-negative. One of the convincing findings in such patients may be subretinal infiltrates detected with B-scan OCT images. We and others reported histopathology of enucleated eyes with VRL, showing lymphoma cell infiltration beneath the RPE layer [8,15]. According to previous reports, subretinal infiltrates are considered to take place in the sub-RPE space, which is recognized as sub-RPE deposits and/or RPE detachments on OCT [11,16,17]. In fact, our case series verified that three of four CB-negative cases with VRL, first suspected of mainly from subretinal infiltrates, subsequently showed favorable responses to IV-MTX. Importantly, such CB-negative VRL cases had milder vitreous haze, which allowed for high-quality OCT images to detect the sub-RPE lesions. In concert with other laboratory assessments such as IL and IgH tests, clinical findings based especially on OCT may well help reach the definite diagnosis of VRL even with CB-negative results. 

VRL-suspicious patients who tested negative for pathological analyses should thus be managed carefully when characteristic OCT findings are observed. Challenging cases are as follows: In VRL-suspicious patients with bilateral involvement, even if informative OCT findings are not obtained from one eye with CB/IL/IgH triple-negative results, diagnostic vitrectomy should be considered in the contralateral eye. If such a patient shows unilateral involvement, ophthalmologists should keep careful observation. Vitreous sampling should be considered when the ophthalmological findings, especially vitreous haze, deteriorate in the unilateral eye.

Myd88 plays an important role in the tumor growth in DLBCL, which is molecular diagnostic target for VRL [18]. Recently, detection of Myd88 mutation on L265P contributed to high sensitivity for the diagnosis of VRL using the vitreous fluids and aqueous humor [19,20]. In this case series, although Myd88 mutation in the intraocular fluids was not performed, it is likely that the mutation test would further increase the diagnostic probability for VRL.

## 5. Conclusions

The diagnostic accuracy of CB cytology in VRL could be equivalent to that of IL and IgH procedures. Subretinal infiltrates might be a potential risk factor to reduce the sensitivity of the CB preparation method. An improved VRL diagnostic rate could be achieved with a combination of CB/IL/IgH pathological assessments as well as OCT findings.

## Figures and Tables

**Table 1:** Diagnostic probability in cell block preparation, interleukin, and immunoglobulin heavy chain gene rearrangement in vitreoretinal lymphoma.

	VRL *n* = 38 Eyes	Uveitis *n* = 7 Eyes	Sensitivity	Specificity
CB	35 (92.1%)	0 (0%)	0.92	1.00
IL	33 (86.8%)	1 (14.3%)	0.87	0.86
IgH	24 (63.2%)	1 (14.3%)	0.63	0.86

CB, cell block preparation; IL, interleukin; IgH, immunoglobulin heavy chain gene rearrangement.

## Data Availability

Data sharing is not applicable.
